# Differential COVID-19 Vaccination Uptake and Associated Factors among the Slum and Estate Communities in Uganda: A Cross-Sectional Population-Based Survey

**DOI:** 10.3390/vaccines11020440

**Published:** 2023-02-14

**Authors:** Joseph Kawuki, Joweria Nambooze, Paul Shing-fong Chan, Siyu Chen, Xue Liang, Phoenix K. H. Mo, Zixin Wang

**Affiliations:** 1 Centre for Health Behaviours Research, Jockey Club School of Public Health and Primary Care, The Chinese University of Hong Kong, Hong Kong, China; 2 Department of Nutritional Sciences and Dietetics, Kyambogo University, Kyambogo, Kampala P.O. Box 1, Uganda

**Keywords:** slum-dwellers, urban poor, COVID-19 vaccination, Uganda

## Abstract

Vaccination against COVID-19 remains one of the ultimate solutions to the ongoing pandemic. This study examined and compared the completion of primary COVID-19 vaccination series and associated factors in the slum and estate communities of Uganda. This was a cross-sectional survey conducted among 1025 slum and estate residents. Logistic regression models were fitted. Of the 1025 participants, 511 were slum residents and 514 were estate residents. Completion of COVID-19 vaccination was 43.8% in the slum community and 39.9% in the estate community (*p* = 0.03). Having more knowledge about COVID-19 was positively associated with completing COVID-19 vaccination in both communities. Perceived benefits and cues to action also had a positive association, but only among the slum residents. However, perceiving people infected with COVID-19 as having a high death rate, perceived barriers such as serious side effects and long distances, and depressive symptoms had negative associations with vaccine uptake among the slum community, but not in the estate community. Addressing barriers to vaccination, strengthening and utilizing the various cues to action, engagement of religious and cultural leaders, and continued community education and sensitization tailored to the needs of each community are potentially vital strategies in raising vaccination rates. Consideration of socioeconomic impact-alleviation strategies, especially among the urban poor, would also be beneficial.

## 1. Introduction

The coronavirus disease 2019 (COVID-19) pandemic remains a major global health crisis, with over 677 million cases and close to 7 million deaths reported worldwide as of 10 February 2023 [1,2]. In Africa, about 13 million cases and 258,000 deaths have been reported, with Uganda having over 170,000 cases and 3630 deaths since the start of the pandemic [1,2]. For context comparison, Uganda has reported similar incidence of COVID-19 cases to its neighbors (Kenya, Rwanda, Burundi and the Democratic Republic of Congo) relative to population size [1]. However, the officially reported COVID-19 cases are non-decisive due to limited testing and surveillance capacities in most developing countries [1,2]. COVID-19 is caused by the severe acute respiratory syndrome coronavirus 2 (SARS-CoV-2), and like other respiratory pathogens, has three primary modes of transmission; contact, droplets, and airborne [3]. In the early stages of the pandemic, various control measures, such as lockdowns, restricted movement, and school closures, among others, were implemented in almost all countries worldwide [4]. Although these measures helped to limit the spread of the virus, they had a dire impact on economies and social lives of individuals, making them unsustainable [4].

With the discovery and approval of vaccines against the disease, infection rates reduced globally, thus enabling several countries to lessen restrictions on movements and lift lockdowns [5,6]. As of 14 December 2022, about 69% of the global population had received at least one dose of COVID-19 vaccines, with over 13 billion doses given [7]. However, distributively, only 25% of people in low-income countries had received at least one dose [7], reflecting evident inequality in vaccination access and uptake [8]. Notably, recent evidence has shown that vaccine acceptance rates vary from country to country, and in Africa, the acceptance rate ranges from 6.9% to 97.9% [9]. The low uptake of COVID-19 vaccination has been attributed to concerns about the adverse effects and effectiveness of the vaccines [9,10]. Intriguingly, the inaccessibility of vaccination centers, global vaccine supply chain dynamics, and perceived misconceptions have been linked to low vaccine uptake, especially in low-income countries [11,12,13]. Nonetheless, vaccination remains the most effective intervention for curtailing communicable disease pandemics [14], and most countries have implemented COVID-19 vaccination programs [7,15]. Uganda rolled out its COVID-19 vaccination program in March 2021, with Oxford–AstraZeneca, Moderna, Sinovac, and Pfizer–BioNTech as vaccine options [16,17]. At first, priority was given to health-care workers and individuals at risk of severe COVID-19, but later universal eligibility for adults was announced in late January 2022 [16,17]. As of 27 November 2022, 41.2% of Uganda’s population had received at least one dose of COVID-19 vaccines, while 27.4% were fully vaccinated [7].

Across countries, the COVID-19 pandemic has shown a disproportionate effect on slum communities. The restrictive measures implemented, especially in the early stage of the pandemic, affected more of the less advantaged, including the urban poor (that is, slum-dwellers) who rely on daily income for survival [16]. In addition, recent studies reported a higher vulnerability to COVID-19 infection and morbidity among slum-dwellers than advantaged or wealthier individuals [18,19]. Disparities in COVID-19 vaccination have been reported among slum-dwellers, with little access to vaccines and several barriers, such as long distances to vaccination centers [16,20].

In Uganda, slum-dwellers are one of the documented high-risk groups, and of the 12 million urban residents in the country, close to 50% live in informal settlements [21]. Residents of informal settlements live in crowded places where social distancing is hardly possible [22,23,24]. In addition, fresh clean water and sanitation for handwashing/hygiene are not easily accessible, and with the low income, buying face masks is also an extra economic burden [23,24,25]. The pandemic worsened the already poor access to health-care services among slum residents in Uganda due to movement restrictions and loss of income/employment [26]. With this vulnerability, prioritizing residents of informal settlements and ensuring adequate uptake of COVID-19 vaccines are paramount, and a clear understanding of the unique factors that may hinder successful vaccination in this group is vital for targeted intervention.

For a more comprehensive evaluation, this study used the socioecological model, as it considers individual and interpersonal level factors. From previous studies, at the individual level, age, gender, education level, income, adequate knowledge and positive perceptions about the COVID-19 virus and vaccines, and history of COVID-19 infection, among others, have been reported to increase the uptake of COVID-19 vaccines in Africa [9,11,13,15]. Interpersonal factors such as place of residence and occupation, among others, have also shown associations with COVID-19 vaccine uptake [13,15]. These potential determinants were considered in this study. Access and exposure to correct information on COVID-19 and vaccination have been shown to affect vaccine uptake [9,27], as they influence people’s knowledge, perceptions and attitudes toward vaccination. Another important factor of interest is the mental health status of individuals. Economic-related mental distress has been reported to have a negative impact on COVID-19 vaccine uptake [28], and given the socioeconomic impact of the pandemic on the urban poor, it was also considered in this study.

Despite the evident vulnerability of the urban poor to the pandemic and the documented associations of COVID-19 vaccine uptake with sociodemographic dynamics, few studies have explored vaccination uptake among this special group. In Uganda, most of the COVID-19 vaccination studies have focused on the general community [11,29,30,31], health-care workers [32,33] and other groups [10], with uncertainty whether the findings apply equally to the disadvantaged urban poor. To our knowledge, only one study has explored COVID-19 vaccination among slum-dwellers, and this reported on the progress of the COVID-19 vaccination rollout in Kampala slums [16]. The study reported generally little vaccine uptake in the first stages of rollout with no gender differences, but with men more likely to be subject to employment-related vaccine mandates, especially market vendors, government workers, and drivers of trucks, taxis, and motorcycles [16]. However, this pilot study did not explore factors associated with COVID-19 vaccine uptake and had no comparable sample to assess the inequities in vaccine uptake. To address these limitations, we conducted a comparative analysis of slum residents and better-off estate residents.

The present study thus aimed to examine and compare the completion of the primary COVID-19 vaccination series and associated factors in the slum and estate communities of Uganda. The estate communities represent a comparable better-off sample, as they tend to have more appropriate housing and better living conditions [34]. Understanding the factors influencing vaccine uptake in these two groups is vital for guiding policy and efforts toward achieving the target vaccination levels for protective herd immunity. We hypothesized that (i) slum residents would have lower COVID-19 vaccine uptake levels than estate residents in formal settlements and (ii) there would be a difference in the factors associated with vaccine uptake in the two groups.

## 2. Materials and Methods

### 2.1. Study Design

**Figure 1:**
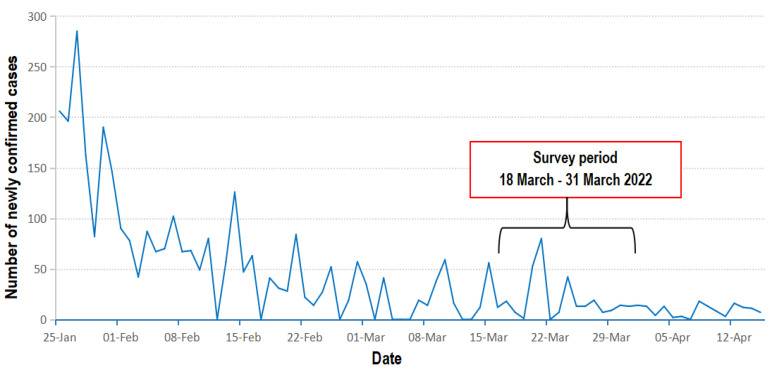
The COVID-19 situation in Uganda during the study period.

This was a cross-sectional survey among slum and estate communities conducted in Kampala and Wakiso districts in Uganda from 18 March to 31 March 2022. Kampala, the capital city, comprises five divisions (Kampala Central, Nakawa, Kawempe, Lubaga and Makindye division) and accommodates more than 3.4 million residents, of which about 50% live in informal settlements/slums [21]. The city houses the largest urban slums in the country, has a population growth rate of 3.2%, and accommodates 45% of all urban residents in Uganda [35]. Wakiso District is located in the Central Region of Uganda, and partly encircles Kampala. Wakiso is the second-wealthiest district in Uganda and houses most of the urban middle-class residents of the country [36]. During the survey period, two peaks of daily COVID-19 cases were reported on 21 March (80 cases) and 24 March (42 cases), after which daily cases remained stable and below 20 cases, as shown in Figure 1.

### 2.2. Participants and Sample Size Planning

The study participants were residents of slum communities and selected suburb estate communities, aged 18 years and above and could speak English or Luganda. The study excluded those who were not able to communicate effectively with the interviewers and those who had lived in the study areas for less than six months.

Our target sample size was 1000 (500 from each group). We assumed the prevalence of completing the primary vaccination series to be 50% in the slum communities. Such a sample could detect the smallest difference of 8.8% in the prevalence of primary vaccination series completion between the slum and estate communities, given a statistical power of 0.80 and an alpha value of 0.05 (two-sided) (PASS 11.0, NCSS LLC). The same approach of using PASS software for sample size calculation has been documented in other similar studies [27,37].

### 2.3. Data Collection

For the slum communities, 10 out of all 57 (Uganda Bureau of Statistics Enumeration Areas) in Kampala and Wakiso districts were randomly selected using the ballot method. Sampling frame mapping was used to identify eligible households, after which systematic sampling was used to select the eligible households. In addition, 10 suburb estate communities in both Wakiso and Kampala districts were purposively selected for data collection. In both communities (slum and estate communities), approximately 50 households were randomly selected from each of the 10 communities (20 in total). Trained interviewers approached each eligible household and invited one eligible household member to complete the interview. If more than one eligible household member was available for the interview, the one whose last birthday was closest to the interview date was invited to join the study. A similar approach was used in a previous population-based survey [38]. Participants were first briefed about the study objectives, procedures and other details. Respondents were reminded of their right to quit the interview at any time without any consequences and anonymity was guaranteed. Written informed consent was sought.

**Figure 2:**
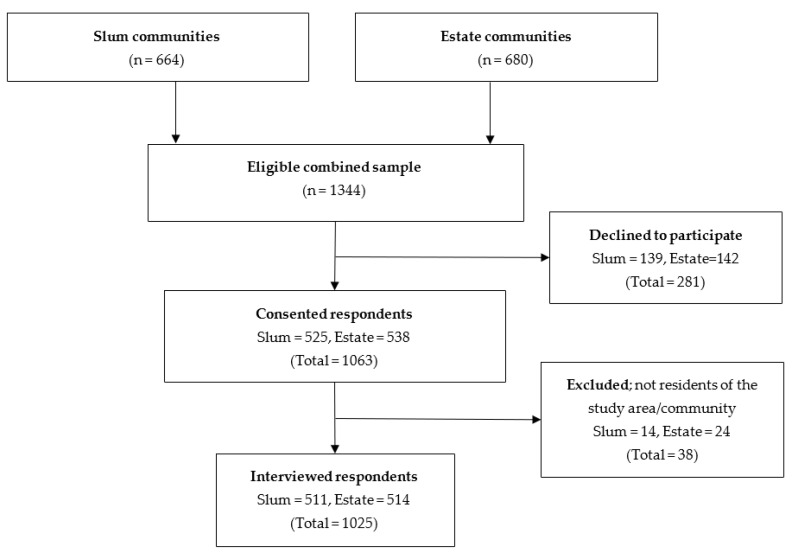
Flowchart of participant selection.

For both communities, data were collected via face-to-face interviews in English and/or Luganda. The interview took about 30 min to complete. A total of 1344 households with eligible respondents were reached, of which 319 declined to participate due to various reasons, yielding a response rate of 76.3% (76.9% for the slum communities and 75.6% for the estate communities) (Figure 2). Ethics approval was obtained from the Clarke International University Institutional Review Board (CLARKE-2021–272) and the Survey and Behavioral Research Ethics Committee of the Chinese University of Hong Kong (SBRE-21-0148).

### 2.4. Measures

#### 2.4.1. Development of the Questionnaire

The questionnaire was developed by a panel of three public health researchers and a health psychologist. Bilingual researchers with master’s degrees translated the English version of the questionnaire to Luganda. The agreed-upon version was back-translated into English by independent bilingual researchers to ensure linguistic equivalence. The questionnaire was then pilot-tested among 27 slum and estate residents of Uganda to assess its clarity and appropriateness. From the pilot study, participants believed that the length was acceptable and the contents easy to comprehend. These 27 participants did not participate in the actual survey. The panel finalized the questionnaire.

#### 2.4.2. Background Characteristics

Participants reported their sociodemographic characteristics (age, gender, education level, monthly household income, marital status, employment status, religion, and tribe), possession of electricity and piped water, whether they shared toilets with other households, presence of chronic disease and COVID-19 infection history.

#### 2.4.3. COVID-19 Vaccination Uptake

Participants reported the number of doses of COVID-19 vaccine received. We defined completion of the primary COVID-19 vaccination series as receiving two doses of inactivated or mRNA vaccines.

#### 2.4.4. Knowledge and Perceptions of COVID-19 Vaccines

Seven items were used to measure participants’ knowledge about COVID-19. Six of these items were adapted from validated measurements in previous studies [37]. We added one item: “The most vulnerable group to severe COVID-19 are the elderly and those with chronic diseases.” The number of correct responses to these items was summed, with a higher score indicating better knowledge of COVID-19.

Regarding perceptions about COVID-19 vaccination, we added one new item—“How high is your chance of having close contact with people with COVID-19?”—to a validated item measuring perceived susceptibility to COVID-19 [37,39], and formed a perceived susceptibility scale. We adapted a five-item perceived benefit scale validated in the Chinese general population to measure the perceived benefit of COVID-19 vaccination [27]. The name “China” was replaced by “Uganda.” We removed one item related to the concern about the cost of COVID-19 vaccination from a validated four-item scale measuring perceived barriers to taking up COVID-19 vaccination, as Uganda was offering free vaccination [27]. We added one item—“Community leaders suggest you take up COVID-19 vaccines”—to the two-item validated scale measuring perceived cues to action related to COVID-19 vaccination [27], as community leaders are considered significant others in Uganda. We used a validated item to measure perceived self-efficacy to receive COVID-19 vaccination without any modification [27,39]. The Cronbach’s alpha of these scales ranged from 0.68 to 0.78.

#### 2.4.5. Mental Health Status

We examined the mental health status of respondents considering the presence of depressive symptoms and anxiety. The validated Patient Health Questionnaire 9 (PHQ-9) scale and the Generalized Anxiety Disorder 7 (GAD-7) scale were used to measure depressive and anxiety symptoms. Cronbach’s alpha for the PHQ-9 and the GAD-7 was 0.89 and 0.91, respectively.

#### 2.4.6. Difficulty Accessing COVID-19 Information and Frequency of Information Exposure

Participants’ difficulty in accessing COVID-19-related information was assessed with a six-item scale constructed for this study (Cronbach’s alpha: 0.89), which had questions on accessing COVID-19 information regarding signs and symptoms, statistics, treatment, personal preventive measures, vaccination, and government policies. We used validated items exploring the frequency of exposure to COVID-19-specific information through different channels, such as web-based media, local channels, health-care workers, and family and friends [40].

### 2.5. Statistical Analysis

The frequency distribution of all studied variables is presented. Differences in characteristics between the slum and estate groups were compared using chi-squared tests (for categorical variables) or independent-sample t-tests (for continuous variables). After controlling for background characteristics with significant between-group differences, the differences in COVID-19 vaccination uptake and independent variables of interest were compared using logistic or linear regression models. Subsequent analysis was performed among slum or estate residents. Completion of the primary COVID-19 vaccination series was the dependent variable. Bivariable logistic regression was then fitted to assess the significance between background characteristics and the dependent variable in the two groups. A single multiple logistic regression model was then fitted, including all significant background characteristics and one independent variable of interest at a time. Crude odds ratios (ORs), adjusted ORs (AORs) and their 95% confidence intervals (CIs) were obtained. We used SPSS 26.0 (IBM Corp., Armonk, NY, USA) for data analysis, with *p* < 0.05 taken as statistical significance.

## 3. Results

### 3.1. Characteristics of the Participants

Of the 1025 participants, 511 were slum residents and 514 residents of estates. The majority of them were below 40 years of age (slum residents 67.7%, estate residents 62.4%), female (slum residents 72.4%, estate residents 69.5%), married or cohabiting with a partner (slum residents 61.4%, estate residents 64.6%), and with full-time, part-time, or self-employment (slum residents 67.5%, estate residents 68.3%). Compared to estate residents, slum residents were younger (*p* = 0.001), less likely to have secondary or above education (48% versus 68.3%, *p* < 0.001), piped water (75.1% versus 91.1%, *p* < 0.001) and electricity (90.0% versus 95.5%, *p* < 0.001) in their households, but more likely to have a monthly household income of 300,000 or below (68.9% versus 28.8%, *p* < 0.001) and shared toilet with other households (91.8% versus 77.2%, *p* < 0.001), (Table 1).

Regarding compliance with personal preventive measures, slum residents were less likely to practice handwashing/hygiene (38.4% versus 44.9%, *p* = 0.02), maintain social distancing (19.4% versus 36.0%, *p* ˂ 0.001), avoid group/social gatherings (25.6% versus 44.6%, *p* ˂ 0.001), and avoid crowded places (31.1% versus 49.4%, *p* ˂ 0.001) than estate residents (Table 1).

### 3.2. COVID-19 Vaccination Uptake

The prevalence of completion of the primary COVID-19 vaccination series among the slum residents was 43.8%, while that among the estate residents was 39.9%. The difference in completion of the vaccination series was statistically significant between the two groups (*p* = 0.03), as shown in Table 2.

### 3.3. Descriptive Statistics of Independent Variables of Interest

Item responses and scale scores for knowledge, perceptions, mental health, difficulty in accessing COVID-19 information, and exposure to various information channels are shown in Table 2. Compared to estate residents, higher proportions of slum residents knew that COVID-19 can be transmitted through feces (56% versus 44%, *p* < 0.001), and that currently there is no effective cure for COVID-19 (40.5% versus 38.3%, *p* = 0.03). A bigger proportion of slum residents also perceived COVID-19 vaccines as highly effective in preventing one from getting COVID-19 (73.0% versus 67.7%, *p* = 0.03), believed that taking up COVID-19 vaccination can bring one’s life back to the time before COVID-19 (55.6% versus 48.6%, *p* = 0.04) and that taking up COVID-19 vaccination can contribute to the control of the pandemic in Uganda (84.1% versus 78.6%, *p* = 0.01). Moreover, slum residents were more concerned that the protection of COVID-19 vaccines will only last for a short time (27.8% versus 25.7%, *p* = 0.03). Many agreed that community leaders suggest they take up COVID-19 vaccines (92% versus 87.7%, *p* = 0.01), compared to estate residents (Table 2).

Slum residents had more depressive symptoms (mean ± SD: 5.82 ± 6.10 versus 4.97 ± 5.53, *p* < 0.001) and anxiety (5.1 ± 5.1 versus 4.5 ± 4.8, *p* = 0.01) compared to the estate residents. They were also more likely to face difficulties in accessing information about COVID-19 statistics (27.4% versus 19.5%, *p* = 0.04), but with less difficulty in accessing the information on personal preventive measures (11.5% versus 12.5%, *p* = 0.03). Slum residents also had less exposure to COVID-19-specific information through web-based media (22.1% versus 34.6%, *p* < 0.001), but with more exposure via local channels (66.5% versus 61.5%, *p* = 0.02), (Table 2).

### 3.4. Factors Associated with Complete Primary COVID-19 Vaccination Series among Slum and Estate Residents

Among the slum residents, older age and Batooro tribe had a positive association with the completion of COVID-19 vaccination, but unemployment, Muslim religion and Basoga tribe had negative associations. Among the estate residents, older age, tertiary education and above, higher monthly household income, COVID-19 infection history, compliance with mask wearing and hand hygiene were associated with a higher completion rate of primary vaccination series, while sharing a toilet with other households had a negative association (Table 3).

After adjusting for these significant background characteristics, having more knowledge of COVID-19 (AOR: 1.15 and 1.18, *p* = 0.049 and *p* = 0.03) was associated with higher odds of completing the primary vaccination series in both the slum and estate communities. In addition, perceived benefit (AOR: 1.18, *p* = 0.04) and cues to action (AOR: 1.34, *p* = 0.04) also had a positive association with the outcome variable, but only among the slum residents. However, perceiving COVID-19-infected people as having a high death rate (AOR: 0.64, *p* = 0.03), perceived barriers such as serious side effects (AOR: 0.66, *p* = 0.03) and long distances to vaccination centers (AOR: 0.65, *p* = 0.03), as well as having depressive symptoms (AOR: 0.97, *p* = 0.04) were associated with lower odds of completing the primary COVID-19 vaccination series in the slum community, but not in the estate community (Table 4).

## 4. Discussion

This is one of the first studies comparing COVID-19 vaccination uptake in slums and better-off communities of Uganda. The study used a comparative approach, allowing for the assessment of inequities in vaccination uptake as well as the associated factors in each group. Moreover, we used stratified sampling methods guided by theories and considered determinants at different levels, all of which are strengths. About 40% of the slum residents were fully vaccinated, a prevalence higher than that reported in urban slums of Pakistan (6%) [41]. The comparator study was, however, conducted in the early stages of vaccination rollout in Pakistan, a period when the vaccine supply was a critical challenge, which might explain the difference. The prevalence recorded in the present study is lower than that of several vaccine acceptance studies in slum communities of Bangladesh (72–93%) [25], India (79%) [42], and Brazil (66.6%) [20]. These studies assessed willingness to get vaccinated, but not actual vaccination uptake, which is hindered by various barriers and dynamics regardless of the willingness, thus the observed low prevalence in our study.

Interestingly, the study revealed that slum residents had a higher rate of completion of the primary COVID-19 vaccination series than estate residents (43.8% vs. 39.9%). This finding deviates from our hypothesis and previous studies that have reported slum-dwellers being less likely to access COVID-19 vaccines [16,20]. A possible reason for this could be the vaccination pass requirements that were imposed on essential workers such as market vendors and taxi and motorcycle drivers, among others, during the early stages of vaccine rollout in the country [16], of which slum-dwellers often make up the majority of these informal jobs. In corroboration, study findings also showed that slum residents with full-time/part-time employment were more likely to be vaccinated than their unemployed counterparts. Nonetheless, previous studies have also reported low acceptance of COVID-19 vaccines among the better-educated and wealthier households in sub-Saharan Africa [30].

Slum residents surprisingly had more knowledge about COVID-19, more perceived benefits of getting vaccinated, and cues to action than the estate residents. In the early stages of vaccine rollout in Uganda, awareness campaigns were decentralized and extended to slum communities, with door-to-door visits and megaphones, informing people about vaccination availability in their community [16]. Such campaigns involved community leaders and village health teams and were also extended to shopping centers, marketplaces, and taxi parks. Slum residents in Uganda also perceived vaccination as an opportunity to get out of the lockdown, which had severely affected their daily income and livelihoods [16]. All these might explain the observed difference. In addition, slum residents reported more mental distress in terms of depression and anxiety than estate residents. The restrictive measures against the pandemic disproportionately affected the disadvantaged urban poor, whose livelihoods were the most affected [16,18,23]. With this loss of income/employment and socioeconomic impact, economic-related mental distress is inevitable [28], thus the observed pattern.

Regarding the factors associated with completing the primary COVID-19 vaccination series, having more knowledge of COVID-19 was positively associated with vaccination uptake among the slum and estate communities. The finding is in line with previous studies that have also reported positive associations [9,11]. This is partly because individuals with good knowledge of the disease tend to be more aware of the importance of getting vaccinated and where to get the vaccine. Slum residents with more perceived benefits and cues to action were also more likely to complete COVID-19 vaccination than those with fewer perceived benefits and cues to action. However, those with perceived barriers such as serious side effects and long distances to vaccination centers were less likely to take COVID-19 vaccination. The findings align with previous health belief model (HBM) studies on COVID-19 vaccination, which have also reported a similar pattern [38]. Notably, none of the components of the health belief model could explain vaccine uptake among the estate community. The estate sample in this study had significantly fewer perceived benefits of getting vaccinated, as well as cues to action, which might partially explain the observation. However, these reasons are inconclusive and call for further in-depth investigation.

The findings revealed that slum residents with more depressive symptoms were less likely to be vaccinated against COVID-19 than their normal counterparts. This aligns with previous studies that have also reported a negative impact of depression on COVID-19 vaccine uptake [28,43,44]. Notably, the negative impact of depression on vaccination was significant only among the slum residents, supporting Bendau et al.’s finding of socioeconomic-related mental distress being associated with low vaccine uptake [28]. Arguably, due to depressive symptoms and cognitive impairment, depressed individuals tend to have little access to accurate information on vaccination, and perhaps may be more concerned about the side effects of the vaccine on their condition and medication [45].

Our findings have several practical implications for improving the COVID-19 vaccination program in Uganda. Significant levels of vaccine hesitance still exist in both communities, implying the need to address the existing barriers to vaccination, as well as strengthening and utilizing potential cues to action. Continuous public sensitization about the disease and the vaccination program is still paramount, especially in such times when the pandemic appears to be fading or becoming less severe in Uganda and other countries. This should be tailored to the specific needs of each community. The engagement of religious and cultural leaders in vaccination campaigns and programs might also be another useful strategy, since both religion and tribe were significantly associated with vaccine uptake, as in previous studies [43,46]. In addition, consideration of relief packages to mitigate the socioeconomic impact of the pandemic in overall COVID-19 prevention programs would be beneficial in improving vaccination uptake, as economic-related distress had a ruinous effect on vaccine uptake, especially among the urban poor.

This study had some limitations. First, recall bias existed since the study outcome was self-reported and prone to social desirability. Second, females and unemployed participants were likely to be oversampled because they were more likely to be home at the time of data collection/interviews. The prevalence of vaccination uptake in the slum residents might be underestimated, as having full-time/part-time work was associated with higher COVID-19 vaccination uptake in this group. Third, some measurement tools were constructed for our study because there were no validated tools, although their reliability was acceptable. Fourth, the slum and estate residents were recruited in just two districts of Uganda, and therefore caution should be taken when generalizing the results to the country. In addition, the study’s cross-sectional design does not allow inference and causation establishment, but rather just associations. Despite the limitations, the study findings provide valuable information on COVID-19 vaccination and associated factors in both the slum and estate communities of Uganda.

## 5. Conclusions

This comparative study revealed that slum communities had a higher completion rate of primary COVID-19 vaccination series than the better-off estate communities, but with evident vaccine hesitance in both groups. Current and future COVID-19 vaccination programs should address the existing barriers to vaccination uptake while utilizing potential cues to action, such as using community leaders, health-care workers and family members to urge their loved ones to get vaccinated. Continued community education and sensitization are also essential and should be tailored to the needs of various communities. The engagement of religious and tribal leaders in vaccination campaigns and consideration of relief packages to alleviate the socioeconomic impact of the pandemic, especially among the urban poor, would be beneficial in increasing COVID-19 vaccination in the country.

## Figures and Tables

**Table 1:** Background characteristics of the participants.

	People from Slum Communities (n = 511)	People from Estate Communities (n = 514)	*p-*Values
	n (%)	n (%)	
**Sociodemographic characteristics**			
Age (years)			0.001
18–29	202 (39.5)	158 (30.7)	
30–39	144 (28.2)	163 (31.7)	
40–49	106 (20.7)	94 (18.3)	
50 and above	59 (11.5)	99 (19.3)	
Gender			0.16
Male	141 (27.6)	157 (30.5)	
Female	370 (72.4)	357 (69.5)	
Educational level			˂0.001
Primary or below	266 (52.1)	163 (31.7)	
Secondary	193 (37.8)	207 (40.3)	
Tertiary and above	52 (10.2)	144 (28.0)	
Monthly Household income (US$1 = 3500 UgX)			˂0.001
300,000 or below	352 (68.9)	148 (28.8)	
300,001–700,000	133 (26.0)	216 (42.0)	
700,001–3,000,000	26 (5.1)	136 (26.5)	
Above 3,000,000	0 (0.0)	14 (2.7)	
Marital status			0.16
Currently single	197 (38.6)	182 (35.4)	
Married or cohabiting with a partner	314 (61.4)	332 (64.6)	
Current employment status			0.42
Full-time/part-time/self-employed	345 (67.5)	351 (68.3)	
Unemployed/retired/student/housewife	166 (32.5)	163 (31.7)	
Religion			0.73
Catholic	164 (32.1)	150 (29.2)	
Protestant	109 (21.3)	115 (22.4)	
Moslem	106 (20.7)	100 (19.5)	
Pentecostal Christian	118 (23.1)	132 (25.7)	
Others	14 (2.7)	17 (3.3)	
Tribe			˂0.001
Baganda	224 (43.8)	310 (60.3)	
Banyankole	52 (10.2)	54 (10.5)	
Banyarwanda	16 (3.1)	15 (2.9)	
Basoga	37 (7.2)	32 (6.2)	
Bakiga	11 (2.2)	15 (2.9)	
Banyooro	9 (1.8)	15 (2.9)	
Bagisu	12 (2.3)	13 (2.5)	
Batooro	69 (13.5)	11 (2.1)	
Iteso	12 (2.3)	7 (1.4)	
Others	69 (13.5)	42 (8.2)	
Possess piped water in your household			˂0.001
No	127 (24.9)	46 (8.9)	
Yes	384 (75.1)	468 (91.1)	
Possess electricity in your household			0.001
No	51 (10.0)	23 (4.5)	
Yes	460 (90.0)	491 (95.5)	
Sharing toilet with other households			˂0.001
No	42 (8.2)	117 (22.8)	
Yes	469 (91.8)	397 (77.2)	
**Health conditions**			
History of confirmed COVID-19 infection			0.01
No	470 (92.0)	447 (87.0)	
Yes	41 (8.0)	67 (13.0)	
Presence of chronic diseases			0.06
No	314 (61.4)	341 (66.3)	
Yes	197 (38.6)	173 (33.7)	
**Compliance with personal preventive measures against COVID-19**			
Wearing a face mask when going to public places like workplaces, public transport, market, shops, place of worship, etc.			0.08
Never/sometimes/often	281 (55.0)	259 (50.4)	
Always	230 (45.0)	255 (49.6)	
Washing hands with soap and clean water or sanitizing hands using alcohol-based hand sanitizers			0.02
Never/sometimes/often	315 (61.6)	283 (55.1)	
Always	196 (38.4)	231 (44.9)	
Maintaining a reasonable social distance between you and others in public places			˂0.001
Never/sometimes/often	412 (80.6)	329 (64.0)	
Always	99 (19.4)	185 (36.0)	
Avoiding group/social gatherings whenever possible			˂0.001
Never/sometimes/often	380 (74.4)	285 (55.4)	
Always	131(25.6)	229 (44.6)	
Avoiding crowded places			˂0.001
Never/sometimes/often	352 (68.9)	260 (50.6)	
Always	159 (31.1)	254 (49.4)	

**Table 2:** COVID-19 vaccination uptake and other independent variables of interest between people from slum communities and estate communities.

	People from Slum Communities (n = 511)	People from Estate Communities (n = 514)	Unadjusted *p*-Values	Adjusted *p*-Value
	n (%)	n (%)		
**COVID-19 vaccination uptake**				
Number of doses of COVID-19 vaccination received by the participants			<0.001	0.004
0	144 (28.2)	206 (40.1)		
1	143 (28.0)	103 (20.0)		
2	222 (43.4)	193 (37.6)		
3	2 (0.4)	12 (2.3)		
Completion of primary COVID-19 vaccination series (2 doses)			0.01	0.03
No	287 (56.2)	309 (60.1)		
Yes	224 (43.8)	205 (39.9)		
**Knowledge related to COVID-19, n (%) correct response**				
COVID-19 can be transmitted through droplets of infected individuals	480 (93.9)	482 (93.8)	0.51	0.93
COVID-19 can be transmitted by touching contaminated objects/surfaces	473 (92.6)	478 (93.0)	0.44	0.60
COVID-19 can be transmitted through contact with asymptomatic patients	423 (82.8)	434 (84.4)	0.26	0.94
COVID-19 can be transmitted through contact with feces	289 (56.6)	226 (44.0)	<0.001	<0.001
The most vulnerable group to severe COVID-19 are the elderly and those with chronic diseases	433 (84.7)	428 (83.3)	0.29	0.28
Some people can get infected with COVID-19 but have no signs and symptoms	387 (75.7)	383 (74.5)	0.35	0.30
Currently, there is no effective cure for COVID-19	207 (40.5)	197 (38.3)	0.26	0.03
Number of correct responses to knowledge related to COVID-19, Mean (SD)	5.3 (1.3)	5.1 (1.3)	0.06	0.02
**Perceptions related to COVID-19 and COVID-19 vaccination**				
Perceived susceptibility to COVID-19, n (%) high/very high				
In general, how high is your chance of contracting COVID-19 in the next 30 days?	76 (14.9)	76 (14.8)	0.52	0.80
How high is your chance of having close contact with people having COVID-19	121 (23.7)	109 (21.2)	0.19	0.58
Perceived susceptibility scale ^1^, mean (SD)	0.4 (0.7)	0.4 (0.7)	0.55	0.84
Perceived severity of COVID-19, n (%) agree/strongly agree				
COVID-19 would result in permanent bodily damage among infected people	276 (54.0)	279 (54.3)	0.49	0.52
People infected with COVID-19 have a high death rate	346 (67.7)	351 (68.3)	0.45	0.86
Perceived benefit of COVID-19 vaccination, n (%) agree				
COVID-19 vaccines are highly effective in preventing you from getting COVID-19	373 (73.0)	348 (67.7)	0.04	0.03
Taking up COVID-19 vaccines could reduce your risk of having severe symptoms of COVID-19 or death	433 (84.7)	431 (83.9)	0.38	0.80
Taking up COVID-19 vaccination can bring your life back to the time before COVID-19	284 (55.6)	250 (48.6)	0.02	0.04
Taking up COVID-19 vaccination can contribute to the control of the pandemic in Uganda	430 (84.1)	404 (78.6)	0.02	0.01
Perceived benefit scale ^2^, mean (SD)	2.9 (1.2)	2.8 (1.3)	0.02	0.03
Perceived barrier to receive a COVID-19 vaccination, n (%) agree				
COVID-19 vaccines have serious side effects	271 (53.0)	289 (56.2)	0.17	0.60
Getting vaccinated requires walking long distances and takes a lot of time	241 (47.2)	234 (45.5)	0.32	0.31
The protection of COVID-19 vaccines will only last for a short time	142 (27.8)	132 (25.7)	0.25	0.03
Cues to action related to COVID-19 vaccination, n (%) agree				
Community leaders suggest you take up COVID-19 vaccines	470 (92.0)	451 (87.7)	0.02	0.01
Friends and family suggest you take up COVID-19 vaccines	450 (88.1)	435 (84.6)	0.07	0.13
Health-care workers suggest you take up COVID-19 vaccines	458 (89.6)	444 (86.4)	0.07	0.06
Cues to Action Scale ^3^, mean (SD)	2.7 (0.7)	2.6 (0.8)	0.02	0.01
Perceived self-efficacy related to COVID-19 vaccination, n (%) agree				
Taking up COVID-19 vaccination is completely under your control	481 (94.1)	493 (95.9)	.12	14
**Mental health status**				
Depressive symptoms (score of the PHQ-9 scale ^4^), mean (SD)	5.8 (6.1)	5.0 (5.5)	0.02	0.001
Generalized anxiety symptoms (score of the GAD-7 scale ^5^), mean (SD)	5.1 (5.1)	4.5 (4.8)	0.07	0.01
**Difficult to access COVID-19-related information, n (%) difficult/very difficult**				
Signs and symptoms of COVID-19	103 (20.2)	84 (16.3)	0.07	0.75
COVID-19 statistics in Uganda	140 (27.4)	100 (19.5)	0.002	0.04
Treatment of COVID-19 in Uganda	127 (24.9)	105 (20.4)	0.05	0.33
COVID-19 personal preventive measures	59 (11.5)	64 (12.5)	0.36	0.03
COVID-19 vaccination program in Uganda	73 (14.3)	65 (12.6)	0.25	0.65
Government policies concerning COVID-19	34 (6.7)	40 (7.8)	0.28	0.43
Difficult to Access COVID-19 Information Scale ^6^, mean (SD)	1.1 (1.6)	0.9 (1.6)	0.11	0.55
**Exposure to COVID-19-specific information through different channels, n (%) sometimes/always**				
Web-based media	113 (22.1)	178 (34.6)	<.001	<0.001
Local channels	340 (66.5)	316 (61.5)	0.05	0.02
Health-care workers	227 (44.4)	230 (44.7)	0.48	0.20
Family members and friends	251 (49.1)	257 (50.0)	0.41	0.17

Adjusted *p*-values: adjusted for background variables with *p* < 0.05 in between-group comparison. ^1^ Perceived susceptibility scale, 2 items; Cronbach’s alpha: 0.78, one factor was identified by exploratory factor analysis, explaining 81.8% of the total variance. ^2^ Perceived benefit scale, 4 items; Cronbach’s alpha: 0.68, one factor was identified by exploratory factor analysis, explaining 52.8% of the total variance. ^3^ Cues to action scale, 3 items; Cronbach’s alpha: 0.69, one factor was identified by exploratory factor analysis, explaining 61.9% of the total variance. ^4^ PHQ-9 scale, 9 items; Cronbach’s alpha: 0.89, one factor was identified by exploratory factor analysis, explaining 54.8% of the total variance. ^5^ GAD-7 scale, 7 items; Cronbach’s alpha: 0.91, one factor was identified by exploratory factor analysis, explaining 64.6% of the total variance. ^6^ Difficult to Access COVID-19 Information Scale, 6 items; Cronbach’s alpha: 0.89, one factor was identified by exploratory factor analysis, explaining 64.2% of the total variance.

**Table 3:** Associations between background characteristics and completion of primary COVID-19 vaccination series among people from slum and estate communities.

	People from Slum Communities (n = 511)		People from Estate Communities (n = 514)	
	OR (95%CI)	*p-*Values	OR (95%CI)	*p-*Values
**Sociodemographic characteristics**				
Age (years)				
18–29	Reference		Reference	
30–39	1.55 (1.02–2.39)	0.049	1.74 (1.08–2.79)	0.02
40–49	1.73 (1.07–2.79)	0.02	2.43 (1.42–4.16)	0.001
50 and above	1.67 (0.93–3.00)	0.08	3.60 (2.11–6.12)	<0.001
Gender				
Male	Reference		Reference	
Female	1.03 (0.70–1.53)	0.87	0.99 (0.67–1.45)	0.94
Educational level				
Primary or below	Reference		Reference	
Secondary	0.95 (0.65–1.38)	0.77	1.40 (0.91–2.16)	0.13
Tertiary and above	1.00 (0.55–1.81)	0.99	2.08 (1.31–3.30)	0.002
Monthly household income (US$1 = 3500 UgX)				
300,000 or below	Reference		Reference	
300,001–700,000	0.82 (0.55–1.24)	0.35	0.80 (0.52–1.23)	0.31
700,001–3000,000	1.45 (0.65–3.22)	0.36	1.64 (1.03–2.64)	0.04
Above 3000,000	N/A		1.60 (0.53–4.79)	0.40
Marital status				
Currently single	Reference		Reference	
Married or cohabiting with a partner	0.77 (0.54–1.11)	0.16	0.74 (0.52–1.07)	0.11
Current employment status				
Full-time/part-time/self-employed	Reference		Reference	
Unemployed/retired/student/housewife	0.70 (0.48–0.92)	0.04	0.83 (0.56–1.21)	0.33
Religion				
Catholic	Reference		Reference	
Protestant	0.94 (0.58–1.52)	0.78	0.91 (0.56–1.50)	0.72
Moslem	0.53 (0.32–0.87)	0.01	0.76 (0.45–1.29)	0.31
Pentecostal Christian	0.75 (0.47–1.21)	0.24	1.05 (0.65–1.68)	0.85
Others	0.57 (0.18–1.77)	0.33	0.99 (0.36–2.75)	0.99
Tribe				
Baganda	Reference		Reference	
Banyankole	1.53 (0.83–2.80)	0.17	0.93 (0.51–1.68)	0.81
Banyarwanda	0.60 (0.20–1.77)	0.35	0.73 (0.24–2.19)	0.57
Basoga	0.42 (0.19–0.93)	0.03	1.14 (0.55–2.37)	0.73
Bakiga	0.29 (0.06–1.38)	0.12	0.53 (0.17–1.71)	0.29
Banyooro	1.64 (0.43–6.26)	0.47	0.97 (0.34–2.80)	0.96
Bagisu	0.94 (0.29–3.04)	0.91	0.65 (0.20–2.15)	0.48
Batooro	2.30 (1.32–4.02)	0.003	2.56 (0.73–8.91)	0.14
Iteso	1.31 (0.41–4.19)	0.65	1.10 (0.24–4.98)	0.91
Others	0.66 (0.37–1.15)	0.14	0.81 (0.42–1.59)	0.54
Possess piped water in your household				
No	Reference		Reference	
Yes	1.03 (0.69–1.54)	0.89	1.58 (0.82–3.03)	0.17
Possess electricity in your household				
No	Reference		Reference	
Yes	1.03 (0.58–1.85)	0.92	1.93 (0.75–4.98)	0.17
Sharing toilet with other households				
No	Reference		Reference	
Yes	0.56 (0.30–1.06)	0.07	0.57 (0.38–0.87)	0.01
**Health conditions**				
History of confirmed COVID-19 infection				
No	Reference		Reference	
Yes	1.71 (0.90–3.25)	0.10	2.05 (1.22–3.44)	0.01
Presence of chronic diseases				
No	Reference		Reference	
Yes	1.13 (0.79–1.62)	0.50	1.43 (0.99–2.08)	0.06
**Compliance with personal preventive measures against COVID-19**				
Wearing a face mask when going to public places like workplaces, public transport, market, shops, place of worship, etc.				
Never/sometimes/often	Reference		Reference	
Always	1.39 (0.98–1.97)	0.07	1.88 (1.32–2.69)	0.001
Washing hands with soap and clean water or sanitizing hands using alcohol-based hand sanitizers				
Never/sometimes/often	Reference		Reference	
Always	1.27 (0.89–1.82)	0.19	1.63 (1.14–2.33)	0.01
Maintaining a reasonable social distance between you and others in public places				
Never/sometimes/often	Reference		Reference	
Always	1.03 (0.66–1.60)	0.89	1.12 (0.78–1.62)	0.55
Avoiding group/social gatherings whenever possible				
Never/sometimes/often	Reference		Reference	
Always	0.94 (0.63–1.41)	0.77	1.29 (0.90–1.83)	0.16
Avoiding crowded places				
Never/sometimes/often	Reference		Reference	
Always	0.84 (0.57–1.23)	0.37	1.20 (0.85–1.71)	0.30

OR: crude odds ratios.

**Table 4:** Factors associated with completion of primary COVID-19 vaccination series among people from slum and estate communities.

	People from Slum Communities (n = 511)		People from Estate Communities (n = 514)	
	AOR (95%CI)	*p-*Values	AOR (95%CI)	*p-*Values
**Knowledge related to COVID-19**				
Number of correct responses to knowledge related to COVID-19	1.15 (1.01–1.32)	0.049	1.18 (1.02–1.37)	0.03
**Perceptions related to COVID-19 and COVID-19 vaccination**				
Perceived susceptibility scale	0.89 (0.69–1.17)	0.41	0.90 (0.68–1.18)	0.44
Perceived severity				
COVID-19 would result in permanent bodily damage among infected people	1.22 (0.84–1.79)	0.30	1.01(0.68–1.49)	0.96
People infected with COVID-19 have a high death rate	0.64 (0.43–0.96)	0.03	1.22 (0.79–1.88)	0.38
Perceived benefit scale	1.18 (1.01–1.38)	0.04	1.13 (0.98–1.32)	0.10
Perceived barriers				
COVID-19 vaccines have serious side effects	0.66 (0.45–0.96)	0.03	0.99 (0.67–1.48)	0.99
Getting vaccinated requires walking long distances and takes a lot of time	0.65 (0.44–0.96)	0.03	0.82 (0.55–1.21)	0.31
The protection of COVID-19 vaccines will only last for a short time	0.89 (0.58–1.35)	0.58	0.92 (0.59–1.44)	0.70
Cues to action scale	1.34 (1.02–1.77)	0.04	1.00 (0.79–1.28)	0.98
Perceived self-efficacy	1.50 (0.68–3.31)	0.31	0.70 (0.27–1.77)	0.45
**Mental health status**				
Depressive symptoms (score of the PHQ-9 scale)	0.97 (0.95–0.99)	0.04	0.99 (0.96–1.03)	0.70
Generalized anxiety symptoms (score of the GAD-7 scale)	0.97 (0.93–1.00)	0.06	1.00 (0.96–1.04)	0.88
**Difficult to access COVID-19-related information**				
Difficult to Access COVID-19 Information Scale	0.98 (0.87–1.10)	0.67	1.04 (0.93–1.17)	0.51
**Exposure to COVID-19-specific information through different channels, n (%) sometimes/always**				
Web-based media	1.54 (0.97–2.46)	0.07	1.14 (0.74–1.77)	0.54
Local channels	0.89 (0.60–1.33)	0.58	0.76 (0.50–1.17)	0.22
Health-care workers	0.81 (0.56–1.19)	0.29	1.12 (0.74–1.67)	0.60
Family members and friends	0.74 (0.50–1.08)	0.12	1.03 (0.68–1.56)	0.88

AOR: adjusted odds ratios, odds ratios adjusted for significant background characteristics listed in Table 3.

## Data Availability

The data presented in this study are available from the corresponding author upon request. The data are not publicly available, as they contain sensitive personal behavior.

## References

[1] Worldometer (2022). COVID-19 Coronavirus Pandemic. https://www.worldometers.info/coronavirus/.

[2] World Health Organisation Weekly Epidemiological Update on COVID-19—21 September 2022. https://www.who.int/publications/m/item/weekly-epidemiological-update-on-covid-19---21-september-2022.

[3] Choudhary O.P., Singh I., Patra G. (2020). Aerosol transmission of SARS-CoV-2: The unresolved paradox. Travel Med. Infect. Dis..

[4] Onyeaka H., Anumudu C.K., Al-Sharify Z.T., Egele-Godswill E., Mbaegbu P. (2021). COVID-19 pandemic: A review of the global lockdown and its far-reaching effects. Sci Prog..

[5] Fu Y., Jin H., Xiang H., Wang N. (2022). Optimal lockdown policy for vaccination during COVID-19 pandemic. Financ. Res. Lett..

[6] Kansakar S., Dumre S.P., Raut A., Huy N.T. (2021). From lockdown to vaccines: Challenges and response in Nepal during the COVID-19 pandemic. Lancet Respir. Med..

[7] Our World in Data (2022). Coronavirus (COVID-19) Vaccinations. https://ourworldindata.org/covid-vaccinations.

[8] Tatar M., Shoorekchali J.M., Faraji M.R., Wilson F.A. (2021). International COVID-19 vaccine inequality amid the pandemic: Perpetuating a global crisis?. J. Glob. Health.

[9] Ackah B.B., Woo M., Stallwood L., Fazal Z.A., Okpani A., Ukah U.V., Adu P.A. (2022). COVID-19 vaccine hesitancy in Africa: A scoping review. Glob. Health Res. Policy.

[10] Bongomin F., Olum R., Andia-Biraro I., Nakwagala F.N., Hassan K.H., Nassozi D.R., Kaddumukasa M., Byakika-Kibwika P., Kiguli S., Kirenga B.J. (2021). COVID-19 vaccine acceptance among high-risk populations in Uganda. Ther. Adv. Infect. Dis..

[11] Kabagenyi A., Wasswa R., Nannyonga B.K., Nyachwo E.B., Kagirita A., Nabirye J., Atuhaire L., Waiswa P. (2022). Factors Associated with COVID-19 Vaccine Hesitancy in Uganda: A Population-Based Cross-Sectional Survey. Int. J. Gen. Med..

[12] Noushad M., Al-Awar M.S., Al-Saqqaf I.S., Nassani M.Z., Alrubaiee G.G., Rastam S. (2022). Lack of access to COVID-19 vaccines could be a greater threat than vaccine hesitancy in low-income and conflict nations: The case of Yemen. Clin. Infect. Dis..

[13] Lin C., Tu P., Beitsch L.M. (2020). Confidence and receptivity for COVID-19 vaccines: A rapid systematic review. Vaccines.

[14] Hajj Hussein I., Chams N., Chams S., El Sayegh S., Badran R., Raad M., Gerges-Geagea A., Leone A., Jurjus A. (2015). Vaccines through centuries: Major cornerstones of global health. Front. Public Health.

[15] Re V.L., Klungel O.H., Chan K.A., Panozzo C.A., Zhou W., Winterstein A.G. (2021). Global covid-19 vaccine rollout and safety surveillance—How to keep pace. BMJ.

[16] Lines K., Sebbanja J.A., Dzimadzi S., Mitlin D., Mudimu-Matsangaise P., Rao V., Zidana H. (2022). Covid-19 Vaccine Rollout: Challenges and Insights from Informal Settlements. IDS Bull..

[17] Reuters (2022). Covid-19 Vaccination Tracker. https://graphics.reuters.com/world-coronavirus-tracker-and-maps/vaccination-rollout-and-access/.

[18] George C.E., Inbaraj L.R., Chandrasingh S., De Witte L.P. (2021). High seroprevalence of COVID-19 infection in a large slum in South India; what does it tell us about managing a pandemic and beyond?. Epidemiol. Infect..

[19] Malani A., Shah D., Kang G., Lobo G.N., Shastri J., Mohanan M., Jain R., Agrawal S., Juneja S., Imad S. (2021). Seroprevalence of SARS-CoV-2 in slums versus non-slums in Mumbai, India. Lancet Glob. Health.

[20] Aguilar Ticona J.P., Nery Jr N., Victoriano R., Fofana M.O., Ribeiro G.S., Giorgi E., Reis M.G., Ko A.I., Costa F. (2021). Willingness to get the COVID-19 vaccine among residents of slum settlements. Vaccines.

[21] Trending Economics (2022). Uganda-Population Living in Slums. https://tradingeconomics.com/uganda/population-living-in-slums-percent-of-urban-population-wb-data.html#:~:text=Population%20living%20in%20slums%20.

[22] Joseph O. COVID-19: Handwashing Adherence Drop by 93 Percent in Kampala Hotspots. December 2020. https://sph.mak.ac.ug/news/covid-19-handwashing-adherence-drop-93-percent-kampala-hotspots.

[23] Bukuluki P., Mwenyango H., Katongole S.P., Sidhva D., Palattiyil G. (2020). The socio-economic and psychosocial impact of Covid-19 pandemic on urban refugees in Uganda. SSHO..

[24] Patel J.A., Nielsen F.B., Badiani A.A., Assi S., Unadkat V.A., Patel B., Ravindrane R., Wardle H. (2020). Poverty, inequality and COVID-19: The forgotten vulnerable. Public Health.

[25] Patwary M.M., Bardhan M., Al Imran S., Hasan M., Tuhi F.I., Rahim S.J., Newaz M.N., Hasan M., Haque M.Z., Disha A.S. (2022). Psychological determinants of COVID-19 vaccine acceptance among urban slum dwellers of Bangladesh. Front. Public Health.

[26] Nuwematsiko R., Nabiryo M., Bomboka J.B., Nalinya S., Musoke D., Okello D., Wanyenze R.K. (2022). Unintended socio-economic and health consequences of COVID-19 among slum dwellers in Kampala, Uganda. BMC Public Health.

[27] Zhang K.C., Fang Y., Cao H., Chen H., Hu T., Chen Y., Zhou X., Wang Z. (2021). Behavioral intention to receive a covid-19 vaccination among Chinese factory workers: Cross-sectional online survey. J. Med. Internet Res..

[28] Bendau A., Plag J., Petzold M.B., Ströhle A. (2021). COVID-19 vaccine hesitancy and related fears and anxiety. Int. Immunopharmacol..

[29] Echoru I., Ajambo P.D., Keirania E., Bukenya E.E. (2021). Sociodemographic factors associated with acceptance of COVID-19 vaccine and clinical trials in Uganda: A cross-sectional study in western Uganda. BMC Public Health.

[30] Kanyanda S., Markhof Y., Wollburg P., Zezza A. (2021). Acceptance of COVID-19 vaccines in sub-Saharan Africa: Evidence from six national phone surveys. BMJ Open.

[31] Wafula S.T., Mugume I.B., Sensasi B., Okware S., Chimbaru A., Nanyunja M., Talisuna A., Kabanda R., Bakyaita T., Wanyenze R.K. (2022). Intention to vaccinate against COVID-19 and adherence to non-pharmaceutical interventions against COVID-19 prior to the second wave of the pandemic in Uganda: A cross-sectional study. BMJ Open.

[32] Otiti-Sengeri J., Andrew O.B., Lusobya R.C., Atukunda I., Nalukenge C., Kalinaki A., Mukisa J., Nakanjako D., Colebunders R. (2022). High COVID-19 Vaccine Acceptance among Eye Healthcare Workers in Uganda. Vaccines.

[33] Kanyike A.M., Olum R., Kajjimu J., Ojilong D., Akech G.M., Nassozi D.R., Agira D., Wamala N.K., Asiimwe A., Matovu D. (2021). Acceptance of the coronavirus disease-2019 vaccine among medical students in Uganda. Trop. Med. Health.

[34] Braubach M., Savelsberg J., World Health Organization (2009). Social Inequalities and Their Influence on Housing Risk Factors and Health: A Data Report Based on the WHO LARES Database.

[35] Atusiimire L.B., Waiswa P., Atuyambe L., Nankabirwa V., Okuga M. (2019). Determinants of facility based–deliveries among urban slum dwellers of Kampala, Uganda. PLoS ONE.

[36] Rafa M., Moyer J.D., Wang X., Sutton P. (2017). Estimating district GDP in Uganda. SSRN.

[37] Pan Y., Fang Y., Xin M., Dong W., Zhou L., Hou Q., Li F., Sun G., Zheng Z., Yuan J. (2020). Self-reported compliance with personal preventive measures among Chinese factory workers at the beginning of work resumption following the COVID-19 outbreak: Cross-sectional survey study. J. Med. Internet Res..

[38] Wang Z., Fang Y., Yu F.Y., Chan P.S., Chen S. (2022). Governmental Incentives, Satisfaction with Health Promotional Materials, and COVID-19 Vaccination Uptake among Community-Dwelling Older Adults in Hong Kong: A Random Telephone Survey. Vaccines.

[39] Zhang K., Fang Y., Chan P.S., Cao H., Chen H., Hu T., Chen Y., Zhou X., Wang Z. (2022). Behavioral intention to get a booster dose of COVID-19 vaccine among Chinese factory workers. Int. J. Environ. Res. Public Health.

[40] Pan Y., Xin M., Zhang C., Dong W., Fang Y., Wu W., Li M., Pang J., Zheng Z., Wang Z. (2020). Associations of mental health and personal preventive measure compliance with exposure to COVID-19 information during work resumption following the COVID-19 outbreak in China: Cross-sectional survey study. J. Med. Internet Res..

[41] Kazmi T., Abdullah M., Khan A.A., Safdar R.M., Afzal S., Khan A. (2022). COVID-19 vaccination acceptance in underserved urban areas of Islamabad and Rawalpindi: Results from a cross-sectional survey. BMC Public Health.

[42] Bhartiya S., Kumar N., Singh T., Murugan S., Rajavel S., Wadhwani M. (2021). Knowledge, attitude and practice towards COVID-19 vaccination acceptance in West India. Int. J. Community Med. Public Health.

[43] Limbu Y.B., Gautam R.K., Pham L. (2022). The Health Belief Model Applied to COVID-19 Vaccine Hesitancy: A Systematic Review. Vaccines.

[44] Nguyen K.H., Chen S., Morris K., Chui K., Allen J.D. (2022). Mental health symptoms and association with COVID-19 vaccination receipt and intention to vaccinate among adults, United States. Prev. Med..

[45] Cai H., Bai W., Du X., Zhang L., Zhang L., Li Y.C., Liu H.Z., Tang Y.L., Jackson T., Cheung T. (2022). COVID-19 vaccine acceptance and perceived stigma in patients with depression: A network perspective. Transl. Psychiatry.

[46] Choi K., Rondinelli J., Cuenca E., Lewin B., Chang J., Luo Y.X., Bronstein D., Bruxvoort K. (2022). Race/Ethnicity Differences in COVID-19 Vaccine Uptake Among Nurses. J. Transcult. Nurs..

